# Bacterial Outer Membrane Vesicles as Antibiotic Delivery Vehicles

**DOI:** 10.3389/fimmu.2021.733064

**Published:** 2021-09-20

**Authors:** Shannon M. Collins, Angela C. Brown

**Affiliations:** Department of Chemical and Biomolecular Engineering, Lehigh University, Bethlehem, PA, United States

**Keywords:** outer membrane vesicles, antibiotics, antibiotic resistance, drug delivery, Gram-negative bacteria

## Abstract

Bacterial outer membrane vesicles (OMVs) are nanometer-scale, spherical vehicles released by Gram-negative bacteria into their surroundings throughout growth. These OMVs have been demonstrated to play key roles in pathogenesis by delivering certain biomolecules to host cells, including toxins and other virulence factors. In addition, this biomolecular delivery function enables OMVs to facilitate intra-bacterial communication processes, such as quorum sensing and horizontal gene transfer. The unique ability of OMVs to deliver large biomolecules across the complex Gram-negative cell envelope has inspired the use of OMVs as antibiotic delivery vehicles to overcome transport limitations. In this review, we describe the advantages, applications, and biotechnological challenges of using OMVs as antibiotic delivery vehicles, studying both natural and engineered antibiotic applications of OMVs. We argue that OMVs hold great promise as antibiotic delivery vehicles, an urgently needed application to combat the growing threat of antibiotic resistance.

## 1 Introduction

The treatment of bacterial infections continues to be more difficult due to the growing number of antibiotic-resistant organisms and the slow pace of antibiotic discovery. Recently, the United States Centers for Disease Control and Prevention (CDC) reported that annually, almost three million people develop antibiotic-resistant infections in the United States, and more than 35,000 die as a result (1). Gram-negative bacteria, in particular, are extremely difficult to treat with many classes of antibiotics due to their complex, dual-membrane cell envelopes (2). A majority of the CDC’s biggest antibiotic resistant threats are Gram-negative bacteria, including carbapenem-resistant *Acinetobacter* and Enterobacterales, and drug-resistant *Neisseria gonorrhoeae* (1). Gram-negative bacteria have been reported to be responsible for more than 30% of nosocomial infections, including 70% of infections acquired in intensive care units (ICUs) in the United States (3). In order to combat Gram-negative-associated infections, research has focused on developing new types of drugs as well as new delivery strategies to overcome the limitations of currently available drugs.

**Figure 1:**
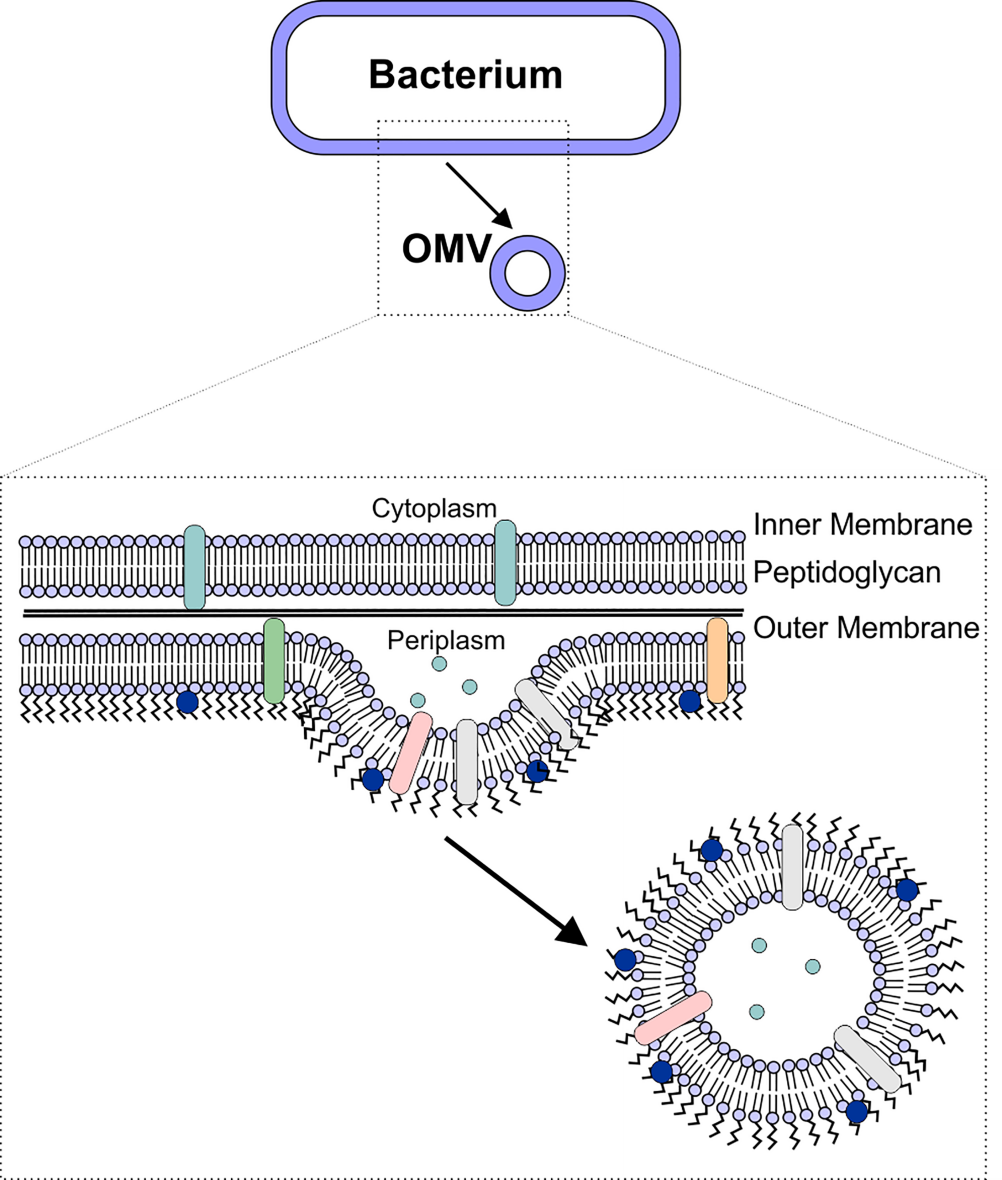
OMV Biogenesis. OMVs are formed due to blebbing of the bacterial outer membrane. The vesicle contains outer membrane-associated proteins and lipids (including lipopolysaccharide), as well as periplasmic components such as peptidoglycan.

Like most other cells, Gram-negative bacteria release membrane vesicles, often referred to as outer membrane vesicles (OMVs) to aid in numerous cellular processes. OMVs are biological spheres that are naturally produced by many, if not all, bacterial species. These bilayered vesicles are derived from the outer membrane of the Gram-negative bacteria, range in size from 50-250 nm in diameter, and contain many of the same components as the outer membrane of the bacterial cell (4–7) (**Figure 1**). In recent years, the role of OMVs in intracellular communication, both between bacterial cells and the host as well as between bacterial cells, has been established (5, 8). This communication is possible due to the ability of the OMVs to deliver a wide range of biomolecules, including proteins, lipids, nucleic acids, peptidoglycan, and small molecules to other cells (8–15). In particular, the unique ability of OMVs to deliver molecules across the Gram-negative cell envelope (10, 14, 16–19) suggests that OMVs have potential as natural antibiotic delivery vehicles to overcome the limitations of antibiotic delivery to these difficult-to-treat bacteria (20–23).

In this paper, we describe the intrinsic delivery functions of OMVs in relation to their potential use as antibiotic delivery vehicles. We provide examples demonstrating successful application of these vehicles for therapeutic purposes and discuss the limitations that remain to be addressed to enable the translation of OMVs as antibiotic delivery vehicles.

## 2 Natural Functions of OMVs

Derived from the outer membrane of Gram-negative bacteria, OMVs contain many similar components, including lipids, proteins, peptidoglycan, and nucleic acids, though not necessarily in the same proportions as in the donor cell (14, 24–27). One of the primary functions of OMVs is to transport these molecules to other cells, including both host and bacterial cells. While much focus has been placed on understanding OMV-mediated virulence factor delivery to host cells to understand the role of OMVs in the host-pathogen interaction, it has become clear that OMVs are also used by bacteria to communicate with neighboring bacterial cells by delivering proteins, genetic material, and quorum sensing molecules. In this section, we describe several specific natural functions of OMVs that provide them with advantages that could be harnessed for the delivery of antibiotics.

### 2.1 Transport Across the Bacterial Cell Envelope

The primary advantage of using OMVs as antibiotic delivery vehicles is their inherent ability to deliver their cargo across the cell envelope of Gram-negative bacteria. With a cell envelope that consists of two membranes, Gram-negative bacteria are inherently resistant to many antibiotics (2). Several reports of the OMV-mediated delivery of active proteins or genes across the Gram-negative cell envelope highlight the potential utility of OMVs to enhance antibiotic delivery to these cells. Although detailed mechanisms of this process remain elusive, future work to better understand this delivery processes will further enhance research into the use of OMVs as antibiotic delivery vehicles to overcome current transport limitations.

**Figure 2:**
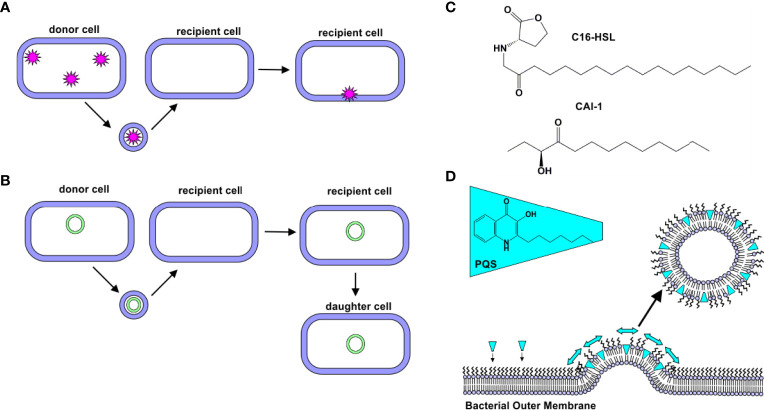
Natural Delivery Functions of OMVs. **(A)** Protein Delivery. Proteins derived from a donor cell are encapsulated within OMVs and delivered to recipient cells. **(B)** Gene Delivery. DNA (plasmid, chromosomal, and/or phage-associated) is encapsulated within OMVs and delivered to recipient cells. In some cases, this new gene is expressed by daughter cells. **(C)** C16-HSL and CAI-1. Hydrophobic quorum sensing molecules, such as C16-HSL and CAI-1, have been observed to be delivered to bacterial cells *via* OMVs. **(D)** Quorum sensing. PQS is a hydrophobic quorum sensing molecule. As it intercalates into the outer leaflet of the bacterial membrane, a wedge-like force promotes the formation of PQS-containing OMVs.

The first reports of OMV-mediated transport across the Gram-negative cell membrane focused on the delivery of peptidoglycan-degrading hydrolases (19, 22). These “predatory” OMVs were hypothesized to fuse with the target cell’s outer membrane, delivering their enzyme cargo to the periplasm of the target cells (22). This hypothesis was supported by subsequent experiments demonstrating that components of *Shigella flexneri* and *Pseudomonas aeruginosa* OMVs are incorporated into the membranes of other bacterial cells (*Salmonella typhi*, *Salmonella typhimurium*, and *Escherichia coli*) (18). More recently, myxobacteria, small Gram-negative soil-dwelling bacteria (28), have been found to produce OMVs encapsulating hydrolytic enzymes (29, 30), which exhibited lytic activity against *E. coli* (14, 31). Together, these findings demonstrate that OMVs are able to deliver cargo to the periplasm of certain bacteria (**Figure 2A**).

In addition to the delivery of intact, functional proteins, OMVs have been observed to facilitate delivery of DNA to bacterial cells (**Figure 2B**). The first evidence of DNA in OMVs was uncovered in 1995, when Kadurugamuwa and Beveridge observed its presence in the OMVs produced by two strains of *P. aeruginosa* (H103 and ATCC 19660) (32). Kolling and Matthews later demonstrated the presence of DNA in OMVs produced by *E. coli* O157:H7. They observed that these OMVs contained DNA encoding certain virulence genes, including *stx1* and *stx2*, which encode for Shiga toxins 1 and 2, respectively. These genes were delivered to noncompetent recipient cells, *E. coli* JM109 (33). Yaron et al. later demonstrated that *E. coli* O157:H7 OMVs transfer DNA to *E. coli* JM109 and *Salmonella* cells, and the recipient cells were shown to express the virulence proteins encoded in the genes (34).

The role of OMVs in the horizontal transfer of antibiotic resistance genes has also been demonstrated. Rumbo et al. observed that two clinical strains of *Acinetobacter baumannii* that were resistant to carbapenem released OMVs containing the *blaOXA-24* gene, which encodes for a β-lactamase. When these *blaOXA-24*-containing OMVs were incubated with a carbapenem-susceptible strain of *A. baumannii*, resistance to several β-lactam drugs was observed. Importantly, this previously susceptible strain was subsequently found to express *blaOXA-24* and to release *blaOXA-24*-containing OMVs (35). Similarly, OMV-mediated horizontal gene transfer has also been identified in the oral pathogen *Porphyromonas gingivalis* (36), *E. coli* O104:H4 (37), and *S. typhi* (38).

Delivery of functional genes and the important role of OMVs in horizontal gene transfer indicates that the OMVs enable delivery of their DNA cargo into the bacterial cytosol. This process was first visualized by Fulsunder et al. by using immunogold labeling of double-stranded DNA to observe movement of DNA from the donor bacterial cells (*Acinetobacter bayli*, JV26) into OMVs and then recipient cells (both *E. coli* DH5α and *A. bayli* JV26) (15).

Together, these observations of OMV-mediated protein and DNA delivery demonstrate that OMVs are able to deliver functional cargo across the Gram-negative cell envelope. This behavior is particularly appealing for the delivery of antibiotics as it suggests that encapsulation of the drugs within OMVs might decrease the transport issues that limit the efficacy of many antibiotics against Gram-negative bacteria.

### 2.2 Delivery of Hydrophobic Molecules

With their membrane structure, OMVs have a unique property of being able to transport both hydrophilic and hydrophobic molecules simultaneously. This property has been demonstrated in the natural delivery processes of OMVs and holds great importance in the future development of OMVs for antibiotic delivery.

Quorum sensing is the process by which bacteria sense cell population density and as a result, alter gene expression. In bacteria, this process occurs through the release of certain molecules; as cell density increases, the concentration of these quorum sensing molecules increases correspondingly, thus serving as a signal of high population density (39). N-acyl-homoserine lactones (AHLs) are the most common quorum sensing molecules employed by Gram-negative bacteria (40). A long-standing question in the quorum sensing field was how hydrophobic, long-chain containing AHLs were delivered through the aqueous extracellular environment. Toyofuku demonstrated that N-hexadecanoyl-L-homoserine lactone (C16-HSL, **Figure 2C**), produced by *Paracoccus denitrificans*, is packaged into outer membrane vesicles to promote solubility of the molecule (41). Similarly, CAI-1, a long-chain ketone QS molecule (**Figure 2C**), was observed to be released in association with OMVs produced by *Vibrio harveyi*. The OMV-associated CAI-1 was able to be delivered in an active form to non-CAI-1-producing cells, including *Vibrio cholerae* (42).

*P. aeruginosa* produces several quorum sensing molecules, including 2-heptyl-3-hydroxy-4-quinolone (*Pseudomonas* quinolone signal, PQS). Mashburn and Whiteley demonstrated that a majority of the produced PQS was released in association with OMVs, while less hydrophobic signaling molecules were not. Interestingly, the authors observed that the PQS molecule itself promotes OMV formation (43). Subsequent work by this group found that PQS intercalates into the outer membrane to induce membrane curvature, thereby promoting OMV formation (42, 44, 45) (**Figure 2D**).

Recently, Choi et al. demonstrated that *Chromobacterium violaceum* delivers the hydrophobic molecule, violacein, to bacterial cells by packaging it in OMVs (46). This process appeared to be regulated, as the OMVs were found to contain more violacein than protein. The OMV-encapsulated violacein retained its activity against the Gram positive organism, *Staphylococcus aureus* (46).

Thus, the natural ability of OMVs to solubilize hydrophobic molecules could enable improved delivery of lipophilic antibiotics, which often exhibit low bioavailability due to poor solubility, limited absorption, and rapid metabolism (47).

### 2.3 Selective Delivery of OMVs to Specific Bacterial Cells

OMVs have been observed to naturally interact with other bacterial cells, both from the same and different species. Selective delivery of their cargo has been observed, but the processes mediating this phenomenon remain unclear.

Tashiro et al. used a classical colloidal science theory, the Derjaguin-Landau-Verwey-Overbeek (DLVO) theory in an attempt to explain the interaction of certain OMVs with specific bacterial cells. This group observed that OMVs produced by *Buttiauxella agrestis* selectively associated with *B. agrestis* cells, enabling delivery of plasmid DNA and gentamicin specifically to *B. agrestis* cells. Because this selective association of OMVs with the bacterial cells did not require the cells to be viable, the authors hypothesized that interaction energies, as calculated using the DLVO theory, might explain this behavior. In this theory, the interaction energy is defined as the sum of the attractive London-van der Waals forces, which depend on OMV radius, and the repulsive electric force, which is a function of the surface charge (zeta potential) of the OMV. The authors observed a correlation between interaction energies and OMV association, which was not entirely linear; therefore, they proposed that this interaction energy is only one factor that regulates OMV specificity for certain bacterial cells, and they hypothesized that surface proteins on both the OMV and bacterial cell surface likely play an additional role in this specific delivery process (48).

Tran and Boedicker investigated whether OMV-mediated DNA transfer is regulated by the relatedness of the OMV donor and recipient cells. The authors observed that *E. coli* is able to encapsulate plasmids with different replication origins within its OMVs and deliver this genetic cargo to recipient cells. *Aeromonas veronii* and *Enterobacter cloacae* exhibited similar behavior. The rate of gene transfer between the three types of OMVs and five types of cells: *E. coli*, *A. veronii*, *E. cloacae*, *C. violaceum*, and *P. aeruginosa* was studied, but no relation between the rates of uptake and the relatedness of the donor and recipient cells was observed (49). The authors did observe that the rates of delivery differed depending on the origin of the OMVs, with *A. veronii* OMVs being the most efficient (49).

Recently, some evidence of the involvement of an OMV surface protein in selective delivery of OMVs was reported. *Agrobacterium tumefaciens* is a phytopathogen that releases OMVs containing a small lipoprotein called Atu8019. The authors of this study observed that OMVs produced by a Δ*Atu8019* deletion mutant were similar in properties to the OMVs released by wildtype cells; however, OMVs from the deletion mutant exhibited an inhibited propensity for cell association, suggesting a role for this protein in selective OMV delivery (50).

The naturally targeted delivery of OMVs to specific bacterial cells holds exciting promise in their development for drug delivery. However, the details of this process have yet to be elucidated. Future research to identify the biological determinants enabling this specificity will enhance the design of targeted delivery systems, both natural and synthetic, for improved antibiotic function.

### 2.4 OMV Stability

A final advantage of using OMVs for antibiotic delivery is their extreme stability and their ability to protect their luminal content from enzymatic degradation, thus promoting long-distance delivery.

The inherent ability of OMVs to protect their cargo from degradation has been widely reported, particularly in the transfer of β-lactamases between bacteria. This process has been observed in a number of organisms, including *A. baumanii*, *Moraxella catarrhalis*, *Stenotrophomonas maltophilia*, *E. coli*, and *P. aeruginosa* (51–55). The luminal location of these antibiotic resistance enzymes has been demonstrated to protect the proteins from enzymatic degradation (54) as well as serum IgG-mediated neutralization (56). In addition, transfer of various protein toxins *via* OMVs has been reported to protect them from enzymatic degradation (12, 57–60), which has been hypothesized to enable long-distance delivery *in vivo* (12).

OMVs are also able to protect their nucleic acid cargo from enzymatic degradation. Koeppen et al. observed that inclusion of RNA within the OMV lumen protected it from RNase digestion (61). Similarly, OMV-encapsulated genes were protected from DNase digestion (34, 35, 62).

In addition to protecting their cargo from enzymatic degradation, OMVs appear to protect their cargo from degradation due to handling and storage. In a systematic study of the stability of OMV-encapsulated cargo, Alves et al. packaged an enzyme, phosphotriesterase (PTE) into the lumen of *E. coli* OMVs. The authors observed increased stability of the protein cargo relative to free PTE against multiple freeze-thaw cycles (63). Later work by this group demonstrated that encapsulation within OMVs protected long-term enzyme activity under multiple storage conditions, including freezing, heating, and lyophilization (64).

Together these observations demonstrate that encapsulation within the OMV lumen is able to protect the cargo from degradation, both *in vivo* and during storage. This property is likely to enhance the activity of encapsulated antibiotics, enabling delivery of reduced dosages.

## 3 Natural Antibiotic Properties of OMVs

OMVs play important roles in the interactions of the microbiota, including interspecies competition. This innate antibiotic property has inspired some groups to propose the use of native OMVs as natural antibiotics (65). These “predatory OMVs” have been observed in many different systems, showing a conservation of this trait across bacterial species.

*P. aerguinosa* OMVs have a well-documented ability to interact with foreign bacteria. Kadurugamuwa and Beveridge observed fusion between native OMVs from strain PAO1 and both Gram-positive and Gram-negative bacteria. Electron micrographs demonstrated the degradation of the bacterial peptidoglycan after incubation with PAO1 OMVs, leading the authors to hypothesize that the OMVs may be carrying autolysins that act to disintegrate the wall of other bacteria cells. Interestingly, the authors found that when the cells were grown in the presence of a sub-inhibitory concentration of gentamicin, the resulting OMVs were even more potent. These antibiotic-loaded OMVs contained less gentamicin than what would normally be used for treatment, but with the added protection from the OMVs, and the additional lytic ability of the OMVs, the antibiotic loaded OMVs were effective in killing the gentamicin-resistant strain, *P. aeruginosa* 8803 (22).

Li et al. investigated the lytic behavior of OMVs produced by 15 different strains of Gram-negative bacteria against 17 different species of Gram-positive and Gram-negative bacteria. They observed significant and broad lytic activity in *P. aeruginosa* PAO1 OMVs, particularly against *E. coli* K12 cells and other cells with similar peptidoglycan structures. Not all OMVs demonstrated lytic activity; those from *Enterobacter agglomerans*, *Klebsiella pneumoniae*, *Citrobacter freundii*, and *Morganella morganii* had very little activity. No OMVs were able to lyse cells of the parent strain (19). This group had previously demonstrated that *P. aeruginosa* OMVs contain a murein hydrolase that is capable of degrading peptidoglycan (66). Additionally, this group has shown that OMVs from *P. aeruginosa* are able to break open the S-layer, the planar paracrystalline structures on some Gram-negative and Gram-positive bacteria that protects the peptidoglycan, and release a peptidoglycan hydrolase (67). They therefore hypothesized that the lytic behavior of the OMVs was due to the presence of these peptidoglycan-degrading enzymes in the OMVs (19).

More recently, OMVs from the soil bacterium, *Myxococcus xanthus*, were demonstrated to lyse *E. coli* cells. The authors observed that the addition of glyceraldehyde-3-phosphate dehydrogenase (GAPDH), an enzyme that enhances membrane fusion, increased the cytotoxicity of the *M. xanthus* OMVs; as a result, the authors concluded that the predatory activity of *M. xanthus* OMVs arises from fusion of the OMVs with the target cell membrane (14). Proteomic analysis demonstrated that *M. xanthus* OMVs contain numerous putative hydrolytic enzymes (30), which may be responsible for this predatory activity. OMVs produced by two additional myxobacterial strains, SBSr073 and Cbv34, were also shown to inhibit *E. coli* growth, a property the authors attributed to the presence of cystobactamids (myxobacterial-derived inhibitors of bacterial gyrase) within the OMVs (68). This group subsequently showed that OMVs produced by CBv34 and Cbfe23 (another myxobacterial strain) were taken up by host cells and inhibited intracellular growth of *Staphylococcus aureus* cells (69).

In addition to encapsulation of anti-bacterial molecules, some OMVs have been observed to naturally encapsulate anti-fungal molecules. Meers et al. demonstrated that *Lysobacter enzymogenes* OMVs exhibit chitinase activity and are able to inhibit the growth of the fungi, *Saccharomyces cerevisiae* and *Fusarium subglutinans*. Importantly, the OMVs were responsible for almost all of the anti-fungal activity of the *L. enzymogenes* supernatant, demonstrating that OMV-mediated transfer of these molecules is the primary pathway for this anti-fungal activity (70).

## 4 Methods of Engineering OMVs to Improve Their Potential as Drug Delivery Vehicles

The ability of OMVs to deliver functional molecules, including proteins, nucleic acids, and small molecules, combined with their natural selectivity has increased interest in their potential as natural delivery vehicles. In particular, OMVs have an intrinsic ability to protect cargo from enzymatic degradation, and with their hydrophobic membrane and hydrophilic lumen, they have the ability to encapsulate a range of drug types. OMVs have been reported to be highly stable (12, 63), and they are readily functionalized to enhance targeted delivery. Despite these numerous advantages, several issues must first be address to enable the translational potential of OMVs as drug delivery vehicles. These challenges include increasing the vesicle yield, reducing the immunogenicity of the OMVs, incorporating specific molecules, and promoting targeting of specific cell types. Continued advancement of these techniques will improve the therapeutic potential of OMVs, particularly as antibiotic delivery vehicles.

### 4.1 Strategies to Increase Vesicle Yield

**Figure 3:**
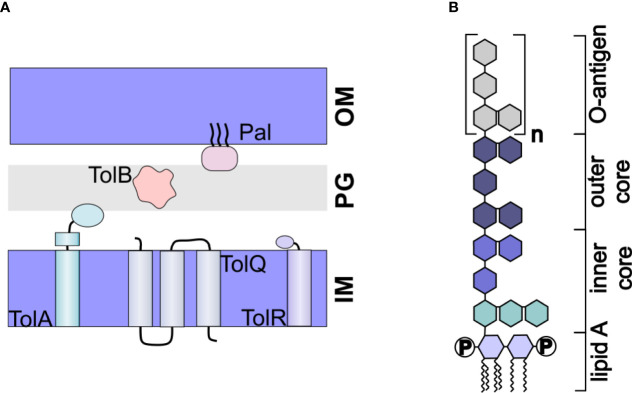
Engineered OMVs. **(A)** The Tol-Pal System. The Tol-Pal system consists of five proteins, TolA, TolQ, and TolR located in the inner membrane (IM), TolB located in the peptidoglycan layer, and Pal located in the outer membrane. Mutation of any of these components has been shown to affect vesiculation. **(B)** Structure of LPS. LPS consists of a hydrophobic lipid A, which is commonly hexaacylated and di-phosphorylated (P). The polysaccharide portion of the molecule consists of a well conserved inner and outer core and a nonconserved O-antigen.

OMVs are naturally produced throughout bacterial growth; however, the resulting yields are too low for biotechnological applications. To overcome this challenge, many groups have looked towards genetic modifications that result in increased vesiculation. Mutations in the Tol-Pal system have been particularly appealing for this purpose. The *tol-pal* operon consists of seven genes, including the five genes comprising the Tol-Pal system: *tolQ*, *tolR*, *tolA*, *tolB*, and *pal*. These genes express proteins that together, form a complex linking the inner membrane, peptidoglycan, and outer membrane (71) (**Figure 3A**). Mutation of any one of the genes results in increased vesiculation in *E. coli* (72, 73). A similar approach that included mutation of the *tolR* gene along with the *galU* gene, which is involved in LPS biogenesis, was found to increase vesiculation in *Shigella sonnei* (74). In *Helicobacter pylori*, deletion of *tolB* but not *pal* increased vesiculation (75).

Building on the finding of the importance of the Tol-Pal system in vesiculation, Henry et al. demonstrated that genetic modifications to promote production of certain protein domains that interact with elements of the Tol-Pal system can likewise increase OMV production. Specifically, they found that periplasmic production of a TolR domain induced a high level of vesiculation in *E. coli*. Additionally, periplasmic production of the translocation domain of colicin A, colicin E3, and minor coat protein g3p also increased vesiculation. Finally, the authors demonstrated that the approach could be used in other bacteria, including *Shigella flexneri* and *Salmonella enterica* (76).

These multiple genetic approaches to increase OMV production have already enabled the use of OMVs for biotechnological purposes, particularly as vaccines and are likely to enable future development of OMVs as natural antibiotic delivery vehicles.

### 4.2 Engineering to Decrease LPS Toxicity

Another important limitation in the use of OMVs as drug delivery vehicles is their inflammatory nature. Derived from the outer membrane, the surface of OMVs is primarily composed of lipopolysaccharide (LPS). LPS consists of a hydrophobic lipid A molecule, which is tethered to the membrane *via* six acyl chains, core oligosaccharides, and the O-antigen (77) (**Figure 3B**). The lipid A portion, also called endotoxin, is responsible for the inflammatory response induced by LPS (78). Thus, to use OMVs as drug delivery vehicles, it is imperative that the toxicity of the LPS be reduced.

One strategy to reduce the toxicity of LPS is to genetically modify the genes leading to full acylation of the lipid A moiety. Nine enzymes are required for the biosynthesis of lipid A (77) and knockout of certain genes encoding these enzymes, in particular *lpxL* and *lpxM* (also known as *msbB*) have been demonstrated to result in under-acylated strains with reduced endototoxicity (79–81).

Alternatively, modification of the phosphorylation of the lipid A moiety can be an effective strategy to reduce endotoxicity of LPS. Lipid A is usually diphosphorylated (82). Edgar Ribi observed that monophosphorylated lipid A is significantly less immunogenic (83), and since then, monophosphoryl lipid A (MPL) has been FDA-approved as an adjuvant (84). A promising strategy to create OMVs consisting of monophosphorylated lipid A is to express the *Helicobacter pylori* Hp0021 gene in the OMV-producing organism. This enzyme removes the 1-phosphate of the lipid A moiety, resulting in monophosphorylated lipid A (85). When this gene was heterologously expressed in *E. coli*, the 1-phosphate group of lipid A was removed (86). While promising, this approach has not yet been used to develop OMVs for biotechnological purposes.

While LPS toxicity remains a serious concern with using OMVs as drug delivery vehicles, multiple promising approaches have demonstrated the potential to reduce the inflammatory response, thus enabling their future use as antibiotic delivery vehicles.

### 4.3 Strategies for Drug Loading

While certain OMVs naturally possess some antibiotic properties, additional work has focused on encapsulating specific molecules within the OMV lumen to expand on the potential of OMVs as delivery vehicles. Several methods to encapsulate different molecules, including antibiotics, have been explored, as described below. Further optimization of these approaches will enable full realization of the potential of OMVs as drug delivery vehicles.

#### 4.3.1 Antibiotic Export *via* OMVs

**Figure 4:**
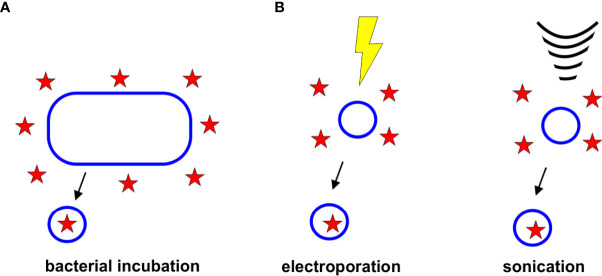
Strategies for Drug Loading. **(A)** Bacterial Incubation. Bacteria grown in the presence of antibiotics have been found to release antibiotic-containing OMVs. **(B)** Electroporation and Sonication. Electroporation and sonication can enhance drug loading within the OMV lumen.

The role of OMVs in removing unwanted material from the cell has been employed as a method to load OMVs with antibiotics. In this passive loading approach, bacterial cells are grown in the presence of the desired drug. The resulting antibiotic-containing OMVs are collected and tested to measure drug loading efficiencies (**Figure 4A**).

Kadurugamuwa and Beveridge first discovered that bacteria grown in the presence of antibiotics release vesicles containing some drug (22). The authors cultured *P. aeruginosa* strain PAO1 in gentamicin and discovered that the OMVs contained 4 ng of drug per μg of protein. These gentamicin-containing OMVs killed *S. aureus*, *E. coli*, and *P. aeruginosa* (strains PAO1 and Pa8803). Importantly, the authors observed that in Pa8803, which is a permeability mutant, OMV encapsulation enhanced gentamicin delivery significantly, demonstrating the potential of OMV-mediated drug delivery to overcome this mechanism of resistance (22). However, the Beveridge group later demonstrated that OMV-mediated delivery is unable to overcome all mechanisms of resistance. The group observed that *Burkholderia cepacia* strain CEP0248 was susceptible to both free and OMV-associated gentamicin, but the highly resistant strain C5424 was not. The OMVs were able to deliver gentamicin; however, the cells were not sensitive to the drug. The authors therefore hypothesized that this strain must possess another mechanism of resistance beyond inhibition of drug uptake (87). Gentamicin-containing *P. aeruginosa* OMVs were also found to be effective in killing some Gram-positive organisms as well, including *Bacillus subtilis* and *S. aureus* (23).

Tashiro et al. hypothesized that the selective delivery of *B. agrestis* OMVs to *B. agrestis* cells (as described in Section 2.4) could enable OMV-mediated selective antibiotic delivery. To test this, the authors grew *B. agrestis* until the late stationary phase, then added gentamicin at a concentration four times higher than the MIC for 30 mins. The purified OMVs were found to contain gentamicin, and selective delivery of gentamicin to *B. agrestis* cells was observed (48).

Huang et al. grew *A. baumanii* in sub-MIC concentrations of levofloxacin and observed that the resulting OMVs contained a high concentration of levofloxacin (20). They demonstrated that OMV encapsulation increased the stability of levofloxacin under a number of storage conditions. The levofloxacin-containing OMVs were able to kill enterotoxigenic *E. coli* (ETEC) cells, and at a low dose, the levofloxacin-containing OMVs were more effective than free levofloxacin. The authors also demonstrated that these levofloxacin-containing OMVs were effective in killing *K. pneumoniae* and *P. aeruginosa* as well, and the procedure for loading could be accomplished with other antibiotics, demonstrating a broad potential of the approach. In a mouse model of ETEC infection, the levofloxacin-containing OMVs were more effective than free drug. Finally, the authors observed that the levofloxacin-containing OMVs were biocompatible (20).

#### 4.3.2 Active Loading Techniques

To enhance the incorporation of drugs and other therapeutics in OMVs and other extracellular vesicles, several active incorporation techniques have been proposed, including electroporation and sonication, (**Figure 4B**). Although none of these techniques have yet been used specifically to load antibiotics into OMVs, it is likely that these approaches could improve the encapsulation efficiencies of antibiotics, as has been observed with other drugs and vesicle types.

##### 4.3.2.1 Electroporation

Electroporation involves the use of a strong electric field to induce the formation of transient pores in a biological membrane (88). This technique has long been used to enhance DNA uptake by bacterial cells (88), and more recently has been used to promote loading of content into human-derived extracellular vesicles (89–93). Gujrati et al. demonstrated that the approach could also be used with OMVs, when they loaded OMVs with siRNA against kinesin spindle protein (KSP) to develop anti-cancer therapeutics. To accomplish loading, the authors electroporated the OMVs in the presence of the siRNA using an empirical approach to identify the optimal conditions (700 V, 50 μF) that promoted loading but did not affect OMV integrity (94). Similarly, Ayed et al. optimized electroporation conditions to load gold nanoparticles (7 nm) into *P. aeruginosa* PAO1 OMVs. The authors observed that one pulse of 0.47 kV was sufficient to encapsulate 55% of the nanoparticles without disrupting OMV integrity (95). These results suggest that electroporation is an effective method for loading a variety of cargo into the OMV lumen, and could have great potential for improving the encapsulation efficiencies of antibiotics.

##### 4.3.2.2 Sonication

Sonication is the use of ultrasonic energy to increase the fluidity of a membrane to enhance drug diffusion. While this technique has not yet been reported as a means of loading drug into bacterial vesicles, it has been used to improve loading efficiencies within human-derived vesicles (96, 97). The primary drawback of this approach is that it may permanently disrupt the integrity of the vesicles (98).

#### 4.3.3 OMV Coating Approach

Wu et al. took advantage of the natural delivery properties of OMVs to enhance delivery of rifampicin-loaded mesoporous silica nanoparticles (MSNs). Rifampicin is a hydrophobic antibiotic with limited effectiveness against Gram-negative bacteria due to its inability to cross the cell envelope. Incorporation of rifampicin within MSNs improves drug solubility, but uptake by Gram-negative bacteria is low. The authors observed that coating the rifampicin-loaded MSNs with *E. coli* OMVs extended the release of drug and the OMV-coated MSNs were taken up by *E. coli* cells more effectively than uncoated MSNs or free drugs. The OMV-coated nanoparticles also displayed good biocompatibility (99).

### 4.4 Surface Engineering

**Figure 5:**
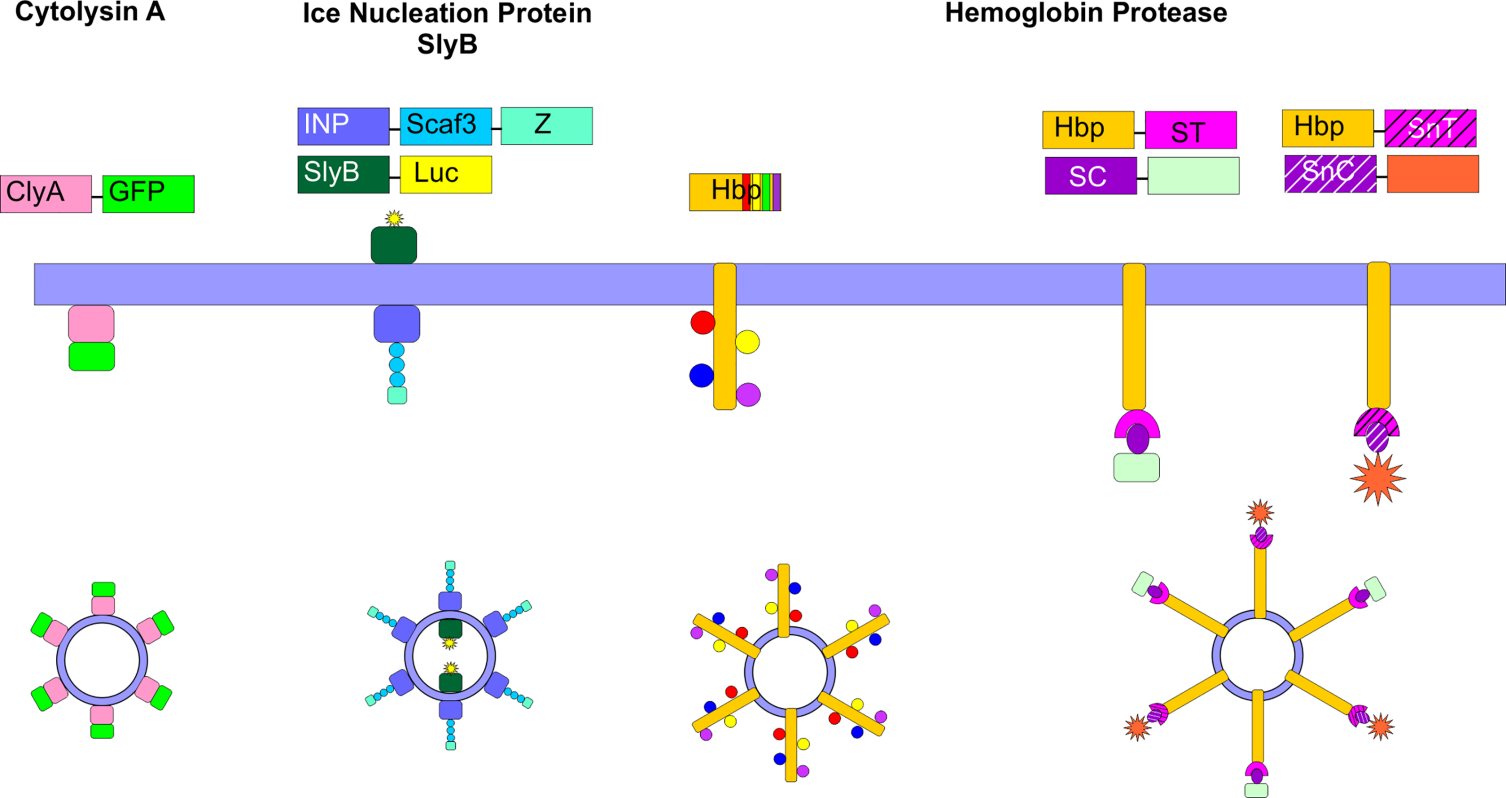
Surface Engineering of OMVs. Several genetic strategies have been used to localize certain proteins on the surface of OMVs. Fusion proteins between cytolysin A (ClyA) and several cargos, including GFP have been created in *E. coli*. Ice nucleation protein (INP) was used to tether an antibody on the surface of the OMV by creating a fusion between INP, a cohesin-containing Scaf3 domain, and an antibody-binding Z-domain. Simultaneously, SlyB was used to localize luciferase to the OMV lumen. Up to four bacterial antigens were tethered to the surface of OMVs using the hemoglobin protease (Hbp). This protein was also used in combination with the SpyCatcher/Tag and SnoopCatcher/Tag systems to display heterologous proteins on the OMV surface.

In order to enhance targeting or functionality of OMVs, several approaches to display particular moieties on the vesicle surface have been employed. These genetic approaches involve the development of fusions between the desired protein and certain surface-localized proteins (42, 100–102), or the use of the SpyCatcher/Tag or SnoopCatcher/Tag systems to promote isopeptide bond formation between the desired protein and a surface-localized protein (103, 104) (**Figure 5**). These approaches have been primarily developed for vaccine technology; however, targeted delivery of antibiotics specifically to pathogenic cells could likely be accomplished using similar approaches.

The ClyA toxin expressed by many *E. coli* strains and enriched in OMVs (27) has been commonly employed as a fusion partner to localize specific proteins to the OMV surface. Kim et al. first demonstrated the power of this approach by creating a series of chimeric ClyA fusion proteins, using green fluorescent protein (GFP), β-lactamase, β-galactosidase, organophosphorous hydrolase (OPH), and a single chain Fv antibody fragment (100). The authors observed that each fusion partner retained its activity and was located on the surface of the vesicles (100). In engineering their siRNA-containing OMVs, Gujrati et al. genetically fused a targeting affibody to the ClyA protein and demonstrated that this approach enabled targeted delivery of the siRNA-containing OMVs to HER2-expressing cells (94).

Another fusion protein approach that has been shown to be successful takes advantage of the autotransporter (AT) Hbp. *Mycobacterium tuberculosis* antigens were localized to the surface of *E. coli* or *Salmonella enterica* OMVs through fusion to Hbp. Hbp is one of the most abundant proteins detected in *E. coli* and *S. enterica* OMVs, making it a strong fusion partner candidate. In addition, the authors exploited the dispensability of certain side domains of Hbp to simultaneously display multiple heterologous antigens on the OMV surface (101). In subsequent work, the authors developed a strategy to overcome limitations in the size and complexity of proteins that can be displayed on the surface of the OMVs *via* fusion to Hbp. In this approach, the authors fused the SpyTag protein (105) to Hbp. Upon translocation of the Hbp across the outer membrane, the SpyTag protein was found to be displayed on the surface of the OMVs. Large protein antigens or nanobodies were then conjugated to the SpyCatcher protein to enable efficient ligation to the OMV. The SnoopTag/SnoopCatcher system (106) was used in tandem to facilitate surface display of heterologous proteins (103, 104).

Chen et al. developed an approach to simultaneously localize proteins of interest within and on the surface of *E. coli* OMVs. To target the OMV lumen, the authors created a fusion protein with SlyB, a native lipoprotein that is localized at the inner leaflet (periplasmic side) of the outer membrane (107). At the same time, they localized an antibody on the surface of the OMV using an ice nucleation protein (INP) anchor (108) tethered to an antibody-binding Z domain *via* a scaffold assembly consisting of 3 cohesin domains (Scaf3). As proof of concept, the authors encapsulated nanoluciferase for detection purposes and tethered IgG to the surface to target thrombin (102).

The ability to functionalize the surface of the OMV to enable selective delivery holds great promise for the targeted delivery of antibiotics. Although not yet commonly employed, targeted delivery of antibiotics represents a promising approach to limit the development of antibiotic resistance, as it would limit exposure of the healthy microbiota to the drug.

## 5 Future Directions

OMVs have several properties that make them promising antibiotic delivery vehicles as described above, including overcoming the entry limitation of certain antibiotics for Gram-negative bacteria. However, while the potential is strong, a number of limitations and challenges remain to be addressed before the use of this novel delivery system can be fully realized.

The mechanisms by which OMVs deliver cargo to bacterial cells remains understudied. While great advances have been made in our understanding of OMV delivery to host cells, little work has focused on delivery to bacterial cells. Additionally, the factors leading to targeted delivery to certain cell types remain unclear. As a more detailed understanding of mechanisms leading to inter-bacterial delivery emerges, researchers will be well-equipped to engineer better performing OMVs or to incorporate specific OMV features into synthetic (liposome) systems to enhance delivery.

In order to realize OMVs as biotechnological devices, several manufacturing advances are necessary. OMV purification remains time-consuming and inefficient, relying on long ultracentrifugation runs as well as filtration and other slow processes. Although OMVs are produced throughout growth, the yield remains low, even for hypervesiculating strains. Thus, more advanced techniques to scale-up these systems are needed. In addition, the heterogeneity of OMVs has recently been established (57, 109–111). For biotechnological applications, it will be essential to develop optimized strategies to purify more homogeneous OMV populations. Furthermore, while some work has demonstrated stability of OMVs under certain storage conditions (63), additional research to identify and/or develop optimal processing and storage conditions that do not affect OMV integrity are necessary.

Finally, standardization of techniques and analyses has been lacking in the OMV field. To overcome this issue, the International Society for Extracellular Vesicles (ISEV) has worked diligently towards developing a set of standards to be applied to all EV studies (112–114). Full adoption of such standards in the OMV field would greatly advance the rate of development of OMVs for biotechnological applications.

## 6 Conclusions

Despite these challenges, the potential of OMVs for antibiotic delivery remains a promising approach to treat Gram-negative bacterial infections, which are otherwise difficult to treat. The field has advanced rapidly over the past 10 years, and it is expected that new discoveries will further advance the biotechnological applications of OMVs, particularly as antibiotic delivery vehicles.

## Author Contributions

SC and AB conceived the concept of the review, and wrote and edited the manuscript. AB prepared the figures, and acquired funding. All authors contributed to the article and approved the submitted version.

## Funding

This research was funded by the National Science Foundation, grant number 1554417 and the National Institutes of Health, grant number DE027769.

## Conflict of Interest

The authors declare that the research was conducted in the absence of any commercial or financial relationships that could be construed as a potential conflict of interest.

## Publisher’s Note

All claims expressed in this article are solely those of the authors and do not necessarily represent those of their affiliated organizations, or those of the publisher, the editors and the reviewers. Any product that may be evaluated in this article, or claim that may be made by its manufacturer, is not guaranteed or endorsed by the publisher.
